# Antiproliferative Activity and Cellular Uptake of Evodiamine and Rutaecarpine Based on 3D Tumor Models

**DOI:** 10.3390/molecules21070954

**Published:** 2016-07-21

**Authors:** Hui Guo, Dongmei Liu, Bin Gao, Xiaohui Zhang, Minli You, Hui Ren, Hongbo Zhang, Hélder A. Santos, Feng Xu

**Affiliations:** 1 Department of Pharmacy, Shaanxi University of Chinese Medicine, Xianyang 712046, China; guohui20032476@163.com (H.G.); liudongmei201605@163.com (D.L.); 2 The Key Laboratory of Biomedical Information Engineering of Ministry of Education, School of Life Science and Technology, Xi’an Jiaotong University, Xi’an 710049, China; bingao0726@163.com (B.G.); xiaohuizhang@mail.xjtu.edu.cn (X.Z.); knightminli@gmail.com (M.Y.); jessie_renhui@aliyun.com (H.R.); 3 Bioinspired Engineering and Biomechanics Center (BEBC), Xi’an Jiaotong University, Xi’an 710049, China; 4 Division of Pharmaceutical Chemistry and Technology, Faculty of Pharmacy, University of Helsinki, Helsinki FI-00014, Finland; hongbo.zhang@helsinki.fi (H.Z.); helder.santos@helsinki.fi (H.A.S.); 5 School of Engineering and Applied Physics, Harvard University, Cambridge, MA 02138, USA; 6 Key Laboratory of Shaanxi Province for Craniofacial Precision Medicine Research, College of Stomatology, Xi’an Jiaotong University, Xi’an 710049, China

**Keywords:** cellular uptake, auto-fluorescence, 3D multicellular spheroids, hanging drop method

## Abstract

Evodiamine (EVO) and rutaecarpine (RUT) are promising anti-tumor drug candidates. The evaluation of the anti-proliferative activity and cellular uptake of EVO and RUT in 3D multicellular spheroids of cancer cells would better recapitulate the native situation and thus better reflect an in vivo response to the treatment. Herein, we employed the 3D culture of MCF-7 and SMMC-7721 cells based on hanging drop method and evaluated the anti-proliferative activity and cellular uptake of EVO and RUT in 3D multicellular spheroids, and compared the results with those obtained from 2D monolayers. The drugs’ IC_50_ values were significantly increased from the range of 6.4–44.1 μM in 2D monolayers to 21.8–138.0 μM in 3D multicellular spheroids, which may be due to enhanced mass barrier and reduced drug penetration in 3D models. The fluorescence of EVO and RUT was measured via fluorescence spectroscopy and the cellular uptake of both drugs was characterized in 2D tumor models. The results showed that the cellular uptake concentrations of RUT increased with increasing drug concentrations. However, the EVO concentrations uptaken by the cells showed only a small change with increasing drug concentrations, which may be due to the different solubility of EVO and Rut in solvents. Overall, this study provided a new vision of the anti-tumor activity of EVO and RUT via 3D multicellular spheroids and cellular uptake through the fluorescence of compounds.

## 1. Introduction

*Evodia rutaecarpa* (Chinese name: Wu-Chu-Yu), a traditional Chinese herb, has been widely used for treating various diseases (e.g., gastrointestinal disorders, amenorrhea, headache, and postpartum hemorrhage) for thousands of years [1]. Evodiamine (EVO) and rutaecarpine (RUT), belonging to the quinazolinocarboline alkaloid class, are the two main bioactive components isolated from Evodiae fructus [2]. Ever since first reported in 1915, the pharmacological activity of these two alkaloids, including anti-inflammatory [3], anti-obesity [4] and anti-tumor effects [5,6,7,8,9,10], has been well studied. For example, it has been reported that EVO has anti-tumor potential by inhibiting cell proliferation and inducing apoptosis via various molecular mechanisms [9,11]. In comparison, the anti-tumor activity of RUT is weak and the underlying mechanism is not clear [12]. Therefore, it is of great importance to further study the anti-tumor effects of EVO and RUT. 

Two-dimensional (2D) in vitro models based on monolayer cell cultures are commonly used to investigate the anti-tumor activity of drugs and identify effective cancer therapies. However, 2D models fail to recapitulate the microarchitecture, cell-cell and cell-matrix interactions, and mass transfer barriers of native tumor tissues [13], which may lead to therapy failure during in vivo trials, and even further in clinical testing [14]. As alternatives, cellular spheroids have emerged as powerful three-dimensional (3D) tissue models for drug development, since cellular spheroids could naturally mimic avascular tumors with inherent metabolic and proliferative gradients to reflect tissue heterogeneity [15,16]. For example, 3D tumor spheroids have been proven to be more physiologically relevant to in vivo tumors for anti-cancer drug testing compared to 2D cultures [14,17]. Various methods have been developed for fabricating 3D tumor spheroids, including hanging drops [18,19], microwells [20] and bioprinting [21,22,23]. Among these methods, the hanging drop method is a scaffold-free technology, with the advantages of easy operation and no need for special equipment [24,25]. Although the antiproliferative and apoptotic effects of EVO and RUT have been well studied on 2D models [26], the application of 3D tumor models for studying their anti-tumor effects has not been explored yet.

**Scheme 1:**
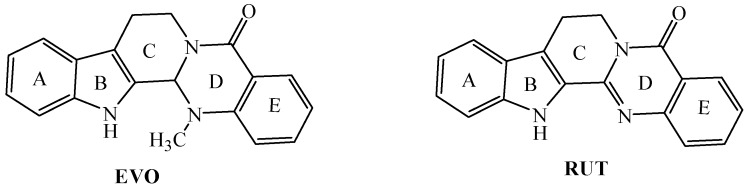
The structures of EVO and RUT.

Quantification of cellular uptake of drugs is very important for assessing their anti-tumor effect [27,28,29], where fluorescence-based methods offer several advantages such as high sensitivity, fast and easy operation [30,31]. EVO and RUT are derivatives of the quino[2′,3′-3,4]pyrrolo-[2,1-*b*]quinazoline ring system whose structures comprise a quinoline ring system, a pyridone ring and a terminal hydroxylactone ring (Scheme 1), similar to the strong topoisomerase I inhibitor luotonin A and anticancer drug camptothecin [32,33]. The rigid planar structure of these alkaloids (containing an extended conjugation of four aromatic and heteroaromatic rings) endows them notable auto-fluorescence, making it possible to track these drugs using fluorescence spectroscopy [34,35]. However, the auto-fluorescence of EVO and RUT has not been explored before for quantitative determination of the cellular uptake of the two drugs.

Herein, for the first time, the anti-tumor activities of EVO and RUT were studied with in vitro 3D tumor spheroid models. In addition, a fluorescence-based method was explored to quantitatively evaluate the cellular uptake of EVO and RUT in MCF-7 and SMMC-7721 cancer cells.

## 2. Results

### 2.1. Fabrication and Characterization of 3D Tumor Spheroids

**Figure 1:**
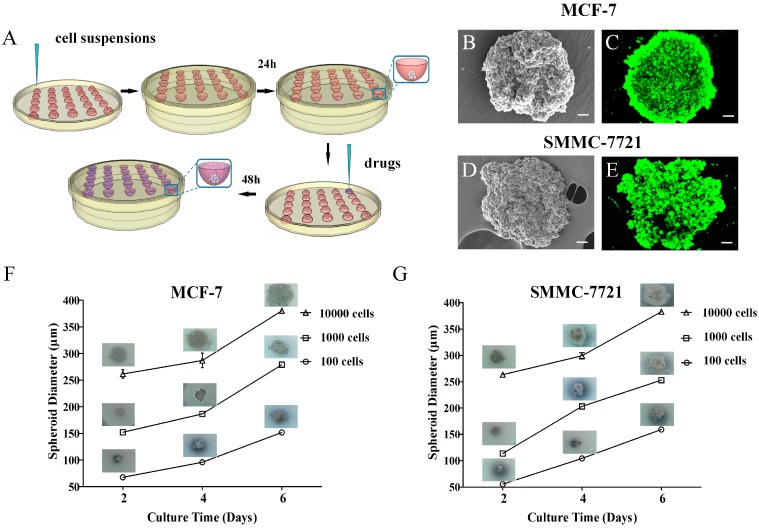
Formation and characterization of 3D tumor spheroids. (**A**) Schematic representation of 3D tumor spheroid formation using a hanging drop method; (**B**) SEM images of MCF-7 spheroids after culture for 2 days and (**C**) live/dead stained fluorescence images of MCF-7 spheroids after culture for 2 days and (**D**,**E**) SMMC-7721 spheroids over 4 days cultures, respectively. Cell growth curves of (**F**) MCF-7 and (**G**) SMMC-7721 cell spheroids with different cell density. Error bars, s.d. (*n* = 3). Scale bars, 20 μm.

To evaluate the anti-tumor effects of EVO and RUT using a native-mimicking model, we first fabricated 3D tumor spheroids of two human cancer cell lines, MCF-7 and SMMC-7721, using a hanging drop method (Figure 1A). From scanning electron microscope (SEM) images, we observed that both MCF-7 and SMMC-7721 cells formed packed spheroid aggregates after culture for 2 days (Figure 1B,D). We also checked the cell viability using live/dead staining and found that most (>90%) of cells in the spheroids remained alive after culture for 4 days, as shown by confocal laser imaging (Figure 1C,E).

To identify appropriate culture conditions, including cell density and cultivation time, for getting suitable 3D tumor spheroids for drug testing, we examined the growth kinetics of the MCF-7 and SMMC-7721 cell spheroids with initial cell numbers of 100, 1000 and 10,000 cells per drop, respectively (Figure 1F,G). Cells in all the groups formed cell spheroids, and the diameter of cell spheroids increased with increasing cultivation time over the period of 6 days for the fixed initial cell numbers. Specifically, the spheroid diameters of MCF-7 with initial cell number of 10,000 were about 261.4 ± 7.9, 287.0 ± 13.7 and 380.1 ± 3.0 μm after culture for 2, 4 and 6 days, respectively, which are similar to those reported in literature [36]. After 2 days of growth, the spheroid diameters of MCF-7 and SMMC-7721 with initially 10,000 cells per drop had already reached 261.4 ± 7.89 and 263.3 ± 2.3 μm, respectively, in which the sizes are clinically relevant for evaluating drug activity [37,38]. Therefore, 10,000 cells per drop and 2 days were selected, respectively, as appropriate culture conditions to achieve the right size of spheroids for subsequent studies [18].

### 2.2. Anti-Tumor Activity Testing of Drugs in 2D and 3D Models

**Figure 2:**
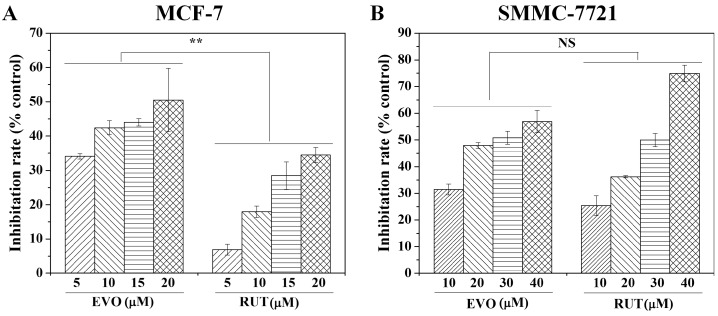
Antiproliferative effects of the model drugs (EVO, RUT) on (**A**) MCF-7 and (**B**) SMMC-7721 cells in 2D model at different drug concentrations after 48 h incubation. The results are expressed as means ± standard deviation (SD) (*n* = 3). ** *p* < 0.01. NS, no significance.

To compare the antitumor activities of EVO with that of RUT in 2D cultures, the in vitro antiproliferative effects of EVO and RUT against MCF-7 and SMMC-7721 were both evaluated via tetrazolium-based colorimetric (MTT) assay. The inhibition rate of drugs to tumor cells was calculated as percentage of dead cells [39]. The inhibition rates of EVO on MCF-7 cells were 34.1% ± 0.6%, 42.5% ± 1.4%, 44.0% ± 0.9%, 53.0% ± 4.1% at drug concentrations of 5, 10, 15 and 20 μM, respectively, while for RUT, they were 6.9% ± 1.3%, 18.0% ± 1.2%, 28.5% ± 3.3%, 34.5% ± 1.8%, respectively (Figure 2A). As for the SMMC-7721 cells, the inhibition rates of EVO were 21.0% ± 1.4%, 47.9% ± 0.8%, 50.8% ± 0.9%, 56.9% ± 1.4% and for RUT were 25.4% ± 3.1%, 36.2% ± 0.3%, 50.0% ± 1.8%, 74.9% ± 2.2%, at drug concentrations of 10, 20, 30 and 40 μM, respectively (Figure 2B). Although the inhibition rates of EVO and RUT on SMMC-7721 cells increased with increasing drug concentrations, the inhibitory rates of EVO were significantly higher than those of RUT for MCF-7 in the concentration range of 5–20 μM (*p* < 0.01), which indicates that EVO may have higher in vitro anti-tumor activity than RUT for MCF-7 cells (Figure 2A). However, there are no significant difference between EVO and RUT for SMMC-7721 in the concentration range of 10–40 μM (Figure 2B), which indicates that EVO and RUT have similar anti-tumor activity to SMMC-7721. We further checked the IC_50_ values of EVO and RUT against the two tumor cell lines after 48 h treatment (Table 1). The IC_50_ values of EVO against MCF-7 and SMMC-7721 cells were 18.1 and 27.4 μM, respectively, and those of RUT were 44.1 and 24.2 μM, respectively.

To validate our 3D tumor models as an effective drug-testing platform, we used CDDP, which is commonly used for testing anti-cancer activity in 3D cancer models [40,41], as a positive control. The IC_50_ concentration of drugs determined in 2D tests (EVO: 18.1 and 27.4 μM for MCF-7 and SMMC-7721, respectively; RUT: 44.1 and 24.2 μM for MCF-7 and SMMC-7721, respectively) (Table 1) were selected as initial drug concentration to evaluate the anti-tumor activities of drugs in the 3D assay. The results showed that the inhibition rates of EVO to MCF-7 and SMMC-7721 tumor cell spheroids were 36.5% ± 1.9% and 39.7% ± 2.1%, respectively, while those of RUT were 27.40% ± 0.52% and 42.20% ± 2.45%, respectively. Since the inhibition rate cannot reach 50% after 48 h of drug treatment in 3D cellular spheroid using the IC_50_ concentration from 2D test, we used drug concentrations of 1-, 2-, 5- and 10-fold the IC_50_ of the drug concentrations from the 2D tests for further 3D studies.

**Figure 3:**
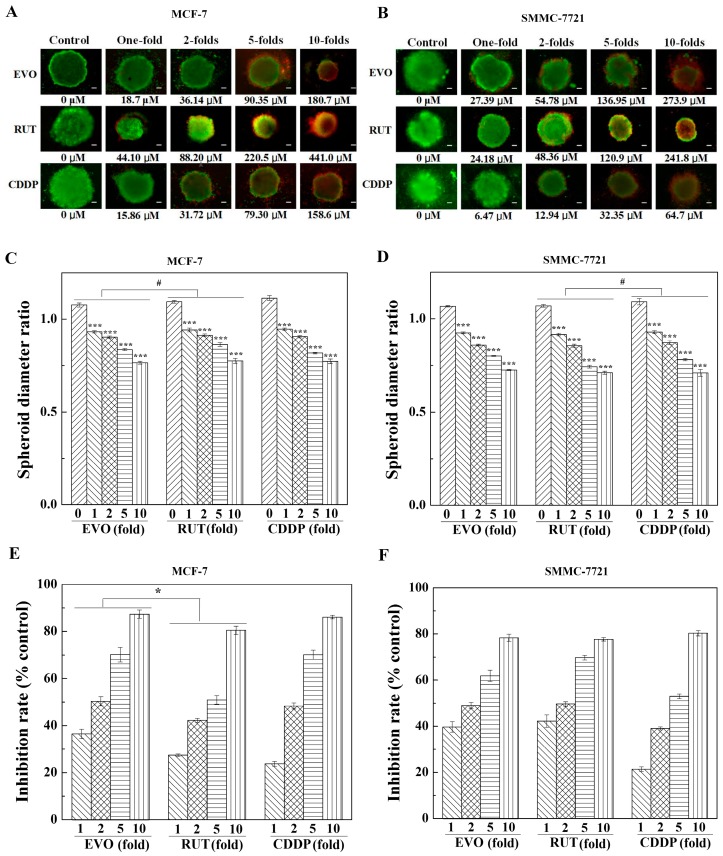
Anti-proliferative effects of the model drugs (EVO, RUT) and control drug (CDDP) on MCF-7 and SMMC-7721 cells in a 3D model. The live/dead images of (**A**) MCF-7 and (**B**) SMMC-7721 spheroids after treatment of different drug concentrations measured. The cell spheroid diameter ratio of (**C**) MCF-7 and (**D**) SMMC-7721 after 48 h treatment of the three drugs at different concentrations. The results are expressed as means ± SD (*n* = 6). *** *p* < 0.001, drugs versus negative control. ^#^ *p* < 0.05. The inhibition rate of the three drugs in (**E**) MCF-7 and (**F**) SMMC-7721 cells in 3D cultures at different concentrations of drug treatment after 48 h incubation. The results are expressed as means ± SD (*n* = 6). * *p* < 0.05. Scale bars, 20 μm.

We first checked the viability of the cells in the spheroids after treatment with drugs at different concentrations by using a live/dead staining assay (Figure 3A,B). We observed that the number of live cells (green) decreased with increasing drug concentration after 48 h treatment. To further check the drug treatment response, we calculated the spheroids diameter after treatment over 2 days, and analyzed the changes of the spheroids diameter ratio (the ratio of spheroid diameters after 48 h of drug treatment relative to that of 0 h) of MCF-7 and SMMC-7721 cells (Figure 3C,D). The spheroid diameter ratio of MCF-7 (Figure 3C) and SMMC-7721 cell (Figure 3D) after 48 h of drug treatment decreased with increasing drug concentration compared with negative control (drug concentration = 0 μM).

Furthermore, the statistical analysis results indicate that there are significant differences (*p* < 0.05) between EVO and RUT for MCF-7 (Figure 3C), and RUT versus CDDP for SMMC-7721 (Figure 3D). To evaluate the anti-tumor activities of EVO and RUT in 3D tumor spheroids, the relationships between drug concentration and the inhibition rate in 3D were studied (Figure 3E,F). The percentage of dead (red) cells of drugs to cell spheroids after 48 h treatment with different concentrations of drugs was calculated from the live/dead staining images. In order to verify the validity of the 3D tumor models we fabricated, CDDP was used as a positive-control drug to study its inhibition rate on MCF-7 spheroids. We observed that CDDP suppressed 23.7% ± 0.9% of the growth of cell spheroids, which is close to the value reported elsewhere [40,41], indicating that the 3D tumor model fabricated herein could be used to evaluate the anti-tumor activity of drugs. The inhibition rate of EVO, RUT and CDDP in MCF-7 spheroids at 1-fold IC_50_ drug concentration was 36.5%, 27.4% and 23.7%, respectively (Figure 3E). At higher drug concentration (e.g., 10-fold the IC_50_), EVO had a similar anti-tumor potential for MCF-7 spheroids as CDDP (EVO: 87.4% at 180.7 μM vs CDDP: 86.2% at 158.6 μM), as shown in Figure 3E. However, RUT was less active than EVO, where it inhibited spheroid growth about 50% at 138.0 μM, which is a much higher value than that of EVO (34.6 μM). Furthermore, the statistical analysis results (Figure 3E) indicate that there is significant difference (*p* < 0.05) between EVO and RUT for MCF-7. These results indicate that EVO may have higher in vitro anti-tumor activity than RUT for MCF-7 cells, as reported elsewhere [39]. However, the statistical analysis results (Figure 3F) indicate that there is no significant difference between EVO and RUT for SMMC-7721, which is consistent with the 2D assay results, indicating EVO and RUT have similar anti-tumor activity for SMMC-7721. To further compare the results of 2D and 3D assays, the IC_50_ values generated from the two assays are shown in Table 1. The IC_50_ of MCF-7 and SMMC-7721 cells in 2D for all three drugs was in the range of 6.4–44.1 μM, while the IC_50_ of the MCF-7 and SMMC-7721 cell spheroids were much higher (21.8–138.0 μM), which may be due to enhanced mass barrier and reduced drug penetration in 3D models.

### 2.3. Cellular Uptake of EVO and RUT in in Vitro 2D and 3D Models

**Figure 4:**
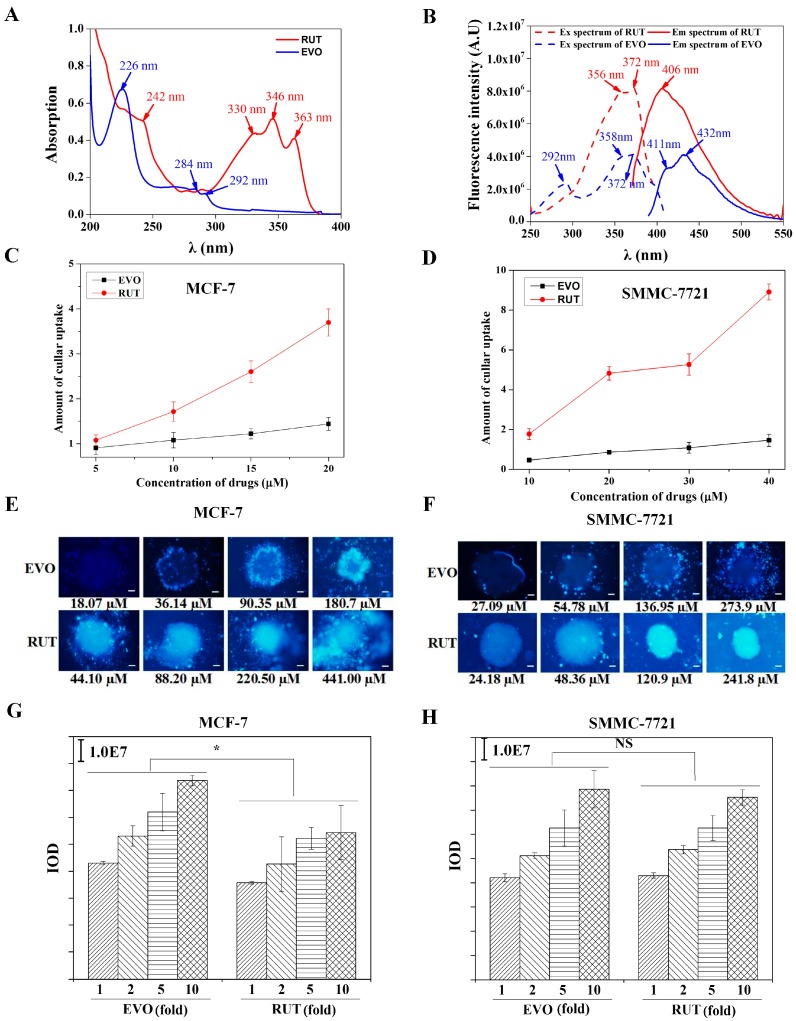
The model drugs, the spectrum characteristics of EVO and RUT and the cellular uptake of the drugs in vitro tumor models. (**A**) UV spectrum of RUT (red line) and EVO (blue line) in CH_3_CH_2_OH (1.0 × 10^−5^ M); (**B**) The fluorescence spectrum of RUT (red line) and EVO (blue line) in CH_3_CH_2_OH (1.0 × 10^−5^ M); The amount of cellular uptake after treatment with the different concentrations of the two drugs in (**C**) MCF-7 and (**D**) SMMC-7721 cells in 2D models; The fluorescence images of 3D (**E**) MCF-7 and (**F**) SMMC-7721 spheroid cultures after treatment with the different drug concentrations measured by fluorescence microscopy. The fluorescence intensity of (**G**) MCF-7 and (**H**) SMMC-7721 cell spheroids by fluorescence microscopy images of the drugs. The results are expressed as means ± SD of six independent experiments. * *p* < 0.05 EVO versus RUT for MCF-7; NS: EVO versus RUT for SMMC-7721. (* *p* < 0.05, NS: no significance).

The rigid planar structure of RUT and EVO containing a quinoline ring system, a pyridone ring and a terminal hydroxylactone ring endows them with notable auto-fluorescence, making it possible to track these drugs using fluorescence spectroscopy. To quantify cellular uptake of EVO and RUT in vitro, we measured the UV/Vis absorption and fluorescence spectra of EVO and RUT in ethanol solutions (10 μM). The UV absorption spectra of RUT (Figure 4A; red line) exhibits an absorption maxima at λ^1^abs = 363 nm, λ^2^abs = 346 nm, λ^3^abs = 330 nm (shoulder) and λ^4^abs = 242 nm (shoulder), and the spectral shape and the position of the emission maxima were close to those described in reference for luotonin A [33]. However, the absorption spectra of EVO (Figure 4A; blue line) exhibited absorption maxima at λ^3^abs = 226 nm, λ^2^abs = 284 nm (shoulder) and λ^1^abs = 292 nm (shoulder), without an absorption in the 300–400 nm region. The fluorescence emission spectra (solid lines) and excitation spectra (dotted lines) of RUT and EVO in ethanol solvent (10 μM) are shown in Figure 4B. We observed that the excitation spectrum of RUT (red dotted lines) exhibited two peaks at 356 nm and 372 nm, which are near to and slightly shifted towards the longer wavelength as compared to the UV-absorption spectra of RUT at 346 nm and 363 nm (Figure 4A), in which the fluorescence maximum excitation (λ_max_) was at 372 nm. With 356 nm and 372 nm as the excitation wavelengths, the emission spectrum of RUT (red solid lines, Figure 4B) consisted only one wide band characterized by a fluorescence maximum at λ_max_ = 406 nm, and was independent of the excitation wavelength. However, the excitation spectra of EVO (blue dotted lines, Figure 4B) showed three resolved peaks at 292, 358 and 372 nm, respectively. With 372 nm as the excitation wavelength, the emission spectra of EVO (blue solid lines, Figure 4B) showed two resolved peaks at 411 and 432 nm, with λ_max_ = 432 nm. Overall, this means that the fluorescence maximum excitation wavelength for both EVO and RUT was 372 nm, but the maximum emission wavelength of EVO was 432 nm, which is an obvious red shift in the fluorescence spectra compared with that of RUT (at 406 nm).

Based on the fluorescence properties of both EVO and RUT, we quantified cellular uptake of drugs through the fluorescence method by using excitation wavelengths of 372 nm, and using emission wavelengths of 406 and 432 nm for RUT and EVO, respectively (Figure 4C,D). The fluorescence intensity of the drugs at different concentrations (0.5, 1.0, 2.5, 5.0, 10.0, 15.0, 20.0 μM) was analysed, and the standard curve was established for the relationship between the concentration of drugs and the fluorescence intensity. In 2D models, the results showed that the cellular uptake of RUT increased with increasing drug concentrations (red lines, Figure 4C,D). However, the EVO concentrations uptaken by the cells showed only a small change increasing drug concentrations (blue lines), and thus, a dose-dependent effect of EVO to two cancer cells was not apparent. For MCF-7 cells, the cellular uptake amount of RUT and EVO in the lowest concentrations (5 µM) is nearly equal, but the lowest concentration of EVO expressed an inhibitory effect which was higher than the effects caused by the same concentration of RUT.

The cellular uptake of drugs in 3D tumor spheroids of MCF-7 (Figure 4E) and SMMC-7721 cells (Figure 4F) were also evaluated through the analysis of the fluorescence intensity of the tumor spheroids based on fluorescence microscopy images of drugs. The results showed that the fluorescence intensity of tumor spheroids increased with increasing drug concentrations (Figure 4G,H). Furthermore, the EVO fluorescence intensity of the tumor spheroids was significantly higher (*p* < 0.05) than that of RUT for MCF-7 (Figure 4G). However, there is no significant difference between EVO and RUT for SMMC-7721 (Figure 4H).

## 3. Discussion

EVO and RUT are the two main bioactive components of a traditional Chinese medicine (namely Evodiae), which have shown their potential anti-tumor activity [42]. However, this has been mainly studied in 2D cancer models. In this study, we aimed to investigate the antiproliferative activity and cellular uptake of EVO and RUT in 3D multicellular spheroids and compare the results with 2D monolayers. Our results from both 2D and 3D studies showed that EVO has higher in vitro anti-tumor activity than RUT for MCF-7 cells, however, for SMMC-7721, they have similar anti-tumor activity. In addition, the drugs’ IC_50_ dramatically increased from the range of 6.4–44.1 μM in 2D monolayers to 21.8–138.0 μM in 3D multicellular spheroids (Table 1). Generally, the chemotherapeutic agents show better potency in 2D models than in 3D models [37]. The missing cell-cell interactions and cell-ECM interactions in 2D models may explain the observed higher anti-tumor activity of drugs such as CDDP [41,43]. Besides, the 3D tissue architecture in spheroids significantly enhances mass barrier, resulting in reduced drug penetration [44]. Although accumulating evidence has shown that there exists significant difference between cell behavior in 2D and 3D, the 3D multicellular spheroid tumor models have been proven to be more physiologically relevant to in vivo tumors [45,46]. An effective 3D in vitro cancer model can provide an efficient way to obtain biological insights that are often lost in 2D platforms. To fully understand the differences in drug resistance of cells in 3D culture vs. 2D, the levels of certain gene expressions of cells should be further studied.

Due to the existence of a conjugated structure, the two compounds of EVO and RUT display notable native fluorescence. This property makes it possible to use fluorescence spectroscopy to determine or predict many biomedical properties. In this study, the cellular uptake of EVO and RUT in tumor models was investigated by using fluorescence method based on the fluorescence properties of the drugs. The results showed that the cellular uptake concentrations increased with increasing drug concentrations for RUT. However, the EVO concentrations uptaken by cells showed only a small change with increasing drug concentrations. The differences in cellular uptake of those two compounds may be due to their molecular structure differences. For example, there is a methyl group on the N atom of EVO but it is absent in RUT, which makes the molecular weight of EVO (MW = 303) being relatively higher than RUT (MW = 289). The solubility of EVO and Rut in different solvents is different [47], which may result in the differences in cellular uptake of these two compounds. For MCF-7 cells, the cellular uptake amount of RUT and EVO in the lowest concentrations (5 µM) are nearly equal, but the lowest concentration of EVO expressed an inhibitory effect, which was higher than the effects caused by the same concentrations of RUT. Wang [9] reported that EVO mediated degradation of estrogen receptor (ER) in MCF-7 cell lines. It suggested that EVO may inhibit breast cancer cell proliferation through the ER-inhibitory pathway. To further understand the differences in activity between these EVO and RUT, the mechanism of drug action needs be studied in the future.

## 4. Materials and Methods

### 4.1. Chemicals and Reagents

All chemicals and reagents were purchased from Sigma-Aldrich Trading Co. Ltd. (Shanghai, China), unless otherwise stated. RPMI 1640 medium, fetal bovine serum (FBS), and penicillin-streptomycin (P/S) were obtained from Gibco (Invitrogen Corporation, Carlsbad, CA, USA). 3-(4,5-Dimethylthiazol-2-yl)-2,5-diphenyltetrazolium bromide was purchased from Thermo Fisher Scientific (Beijing, China).

### 4.2. Cell Culture

SMMC-7721 and MCF-7 cells were purchased from the Type Culture Collection of Chinese Academy of Sciences (Shanghai, China). MCF-7 cells were cultured in RPMI 1640 with 10% (*v*/*v*) FBS. SMMC-7721 cells were cultured in Dulbecco’s Modified Eagle’s Medium (DMEM) with 10% (*v*/*v*) FBS in a humidified atmosphere containing 5% CO_2_ at 37 °C. Cell subcultures were performed by transferring the cell suspension to another culture flask after detachment from the flasks with trypsin. The cell concentration was counted by using a hemocytometer.

### 4.3. Fabrication of 3D Tumor Spheroids

The hanging drop method was used to form 3D spheroids of MCF-7 and SMMC-7721 cell lines (Figure 1A). Firstly, single-cell suspensions were generated from trypsinized monolayers and diluted to the desired cell density. Then, 10 μL drops of cell suspension (1 × 10^6^ cells/mL) were deposited onto the bottom of a culture dish lid, upon inversion of the lid, the 50 hanging drops were formed by surface tension. The lid was placed on the dish and 3 mL PBS buffer solution was filled in the bottom of the dish to avoid over evaporation of the medium. After 24 h of incubation in a humidified atmosphere with 5% CO_2_ at 37 °C, the cells accumulated at the free liquid-air interface.

### 4.4. MTT Assay

Drug sensitivity in 2D culture systems was tested by the MTT assay. When cells reached the period of logarithmic phase, they were trypsinized into single cells with a density of 1 × 10^4^ cells per well in 96-well plates (180 μL/well). After 12 h of incubation, the 20 μL medium containing various concentrations drugs (5, 10, 15, and 20 μM for MCF-7; and 10, 20, 30, and 40 μM for SMMC-7721) or a drug-free medium (control) were added. After 48 h of incubation, the media were removed and washed with PBS, replaced by 200 μL of 0.5 mg/mL MTT solution in RPMI 1640 media. Following 4 h of incubation in this condition, the MTT solution was removed and 200 μL DMSO was added, and the plates were shaken for 10 min. The optical density (OD) of each condition was measured using a microplate reader (Multiskan G0, Thermo Scientific, Rockford, IL, USA) at a wavelength of 570 nm with a reference wavelength of 630 nm. Then, the inhibition rate of drugs to tumor cells was calculated as percentage of dead cells, as described elsewhere [39]. The IC_50_ values were obtained by fitting the data to a sigmoidal dose-response curve (variable slope) using Prism 4 software (GraphPad) [48]. Each experimental condition was repeated for at least three times.

### 4.5. Cellular Uptake of EVO and RUT

Cells were dispensed in 6-well plates at a density of 1 × 10^5^ cells/well (1800 μL/well). After 12 h of incubation, 200 μL of different drugs (5, 10, 15, and 20 μM for MCF-7; and 10, 20, 30, and 40 μM for SMMC-7721) were added into the wells. After 48 h of incubation, the culture medium was removed and the wells were washed with PBS and normal saline for three times. Then, the cells were carefully scraped and dispensed into centrifuge tubes that were placed in an ice water bath for ultrasonication with an ultrasonic cell disruptor. Acetic ether (500 μL) was then added to dissolve cell debris, and the mixture was shaken carefully, followed by extraction of the supernatant liquid. This process was performed in three times and the supernatant liquids were collected. The combined supernatant liquid was volatilized through nitrogen. After drying, 2 mL water was added to dissolve the components, and analyzed with a QuantaMaster 40 spectrofluorometer (Photon Technology International, Lawrenceville, NJ, USA) in a 1 cm quartz cell. Optical excitations were carried out with a 372 nm argon laser beam, and the fluorescence emission intensity was detected at wavelengths of 406 nm and 432 nm for RUT and EVO, respectively. The fluorescence intensity of the drugs at different concentrations (0.5, 1.0, 2.5, 5.0, 10.0, 15.0, and 20.0 μM) was determined and a standard curve was established according to the relationship between the concentration of the drugs and the fluorescence intensity. Based on the standard curve, the cellular uptake amount of both EVO and RUT in the two cancer cell lines was calculated.

### 4.6. Live/Dead Assay

Chemosensitivity in 3D culture system was evaluated by a LIVE/DEAD Viability/Cytotoxicity Kit [18]. Briefly, spheroids were seeded in the lid of the dish for 48 h to reach an average diameter of 250–320 μm. For drug treatment, the media were removed and replaced by 20 μL fresh media with different concentration of drugs at 48 h (final drugs concentration became 1-fold, 2-folds, 5-folds and 10-folds of IC_50_ drug concentration determined in the 2D culture system). CDDP was used as a positive control on each assay. Spheroids were added 10 μL of 2 mM calcein AM and 4 mM ethidium homodimer-1, and incubated for 1 h at 37 °C. The live cells are stained by calcein AM, while the dead cells are stained by ethidium homodimer-1. Images were acquired using a fluorescence microscope (Olympus IX81, Tokyo, Japan) from which the percentages of live (green) and dead (red) cells were quantified. Each experiment was repeated six times.

### 4.7. Morphology, Physical and Optical Characterization

SEM (JSM-6700F, JEOL, Tokyo, Japan) operating at 10 kV was used to characterize cellular morphology. Cellular spheroids were fixed overnight on 2D glass slides with 2.5% glutaraldehyde solution, dehydrated with sequential ethanol solution of 50, 70, 90, 95, and 100%, and then freeze dried. Spheroids were coated by gold sputtering for 120 s and viewed with SEM. UV/Vis spectra were recorded using a UV-3600 UV-Vis-NIR absorption spectrophotometer (Shimadzu Co., Kyoto, Japan), and luminescence spectra with a QuantaMasterTM40 spectrofluorometer in a 1 cm quartz cell at room temperature. The drugs’ luminescence images were acquired using Olympus IX81 fluorescent microscope. The optical density (OD) was measured using Multiskan G0 microplate reader.

### 4.8. Statistical Analysis

All results were presented as mean ± standard deviation (SD). Student’s *t*-test was used to analyze the statistically significant differences between cells exposed to drugs and their controls, where statistical significance was defined as *p*-values less than 0.05 (*p* < 0.05).

## 5. Conclusions

In conclusion, the 3D spheroid assay provides a different potency trend of EVO and RUT compared to 2D monolayer screening. Our findings may provide evidence for explaining why the effect of some anticancer drugs have demonstrated valid effects on cancer cells when evaluated using in vitro 2D cell culture system, but have shown significant discrepancies in the observed efficacy when these drugs are evaluated in vivo. We believe our results may help for drug screening and cytotoxicity studies.

## Figures and Tables

**Table 1:** The IC_50_ values of the model drugs (EVO, RUT) and control drug (CDDP) against the two tumor cell lines in 2D and 3D culture systems at different drug concentrations after 48 h treatment. Unit: μM.

Cells	EVO		RUT		CDDP	
	2D	3D	2D	3D	2D	3D
**MCF-7**	18.1	34.6	44.1	138.0	15.9 [40]	39.9
**SMMC-7721**	27.4	56.2	24.2	42.3	6.4 [40]	21.8
